# Notes and Comments

**Published:** 1894-06

**Authors:** 


### Notes and Comments.[1]

The Independence of Character.—It is easy to live in the world after the world's opinion. It is easy to live in solitude after our own. But the great man is he who, in the midst of the crowd, keeps with perfect sweetness the independence of his character.—*Emerson.*

The Art of Thinking.—In writing upon the subject of thinking and reading, Dr. T. B. Welch says truly, that it is easy and entertaining to read an article which tells you something which you knew before and which you can indorse, but you learn nothing by reading it. It often requires an effort to read an article which contains real information, however plainly expressed. It has to be studied, applied, digested, criticised; the suggestions raised by its perusal have to be followed out to their conclusions; and to conscientiously read an article of this character is a task which some men are inclined to shirk, just as a lazy man might shirk a physical task. But compare the man who shirks with the man who reads, and you will find the first a mental bungler, the second the acute and able thinker, the man whose head saves his hands, and who is valued, respected, and trusted with the conduct of work and administration of affairs, and rewarded accordingly. Always read a little ahead of yourself. Read matter which requires effort on your part to understand. The effort will not only place you on a higher intellectual plane, but the mental exercise will develop a habit of accurate thinking which will be of more value to you than volumes of average matter read only to be forgotten.

[1] The assistant editor solicits contributions for this department,—new methods, new remedies and formulas, or any short practical note which may prove of value to the practitioner or student. Address 212 South Fifteenth Street, Philadelphia.

The Relative Merits of Filling-Material.—A very interesting and practical paper upon the subject of filling-materials was read before the Second District Dental Society of the State of New York by Professor E. T. Darby, and is published in the March issue of the *Dental Cosmos.* After reviewing the history and essential qualities of the different filling-materials, the doctor gives some personal experiences, and closes the essay with the following pertinent remark:

"The ideal filling has not yet been found; but it will surely come. There is no better field for the dental chemist to labor in than this. He may come upon it as by accident, or it may require months or years of patient experimentation, or it may come to him in dreamy visions of the night, as an inspiration. It matters not how. But when he shall have combined in a plastic material adaptability, impermeability, indestructibility, non-conductibility, absence of shrinkage, and harmony of color, then shall his name be engraved upon the pinnacle of fame, and the dental profession throughout the world will rise as one man and call him blessed."

---

Care and Preservation of Eyesight.—Dr. L. Webster Fox, in a lecture published in the *Journal of the Franklin Institute,* discusses eyesight in middle life and old age, and gives certain suggestions for its care and preservation, from which the following notes are made: In addition to the well-known effects of tobacco and alcohol upon the optic nerve, it is pointed out that the drinking of beer and wine leads to changes in the crystalline lens. Deleterious influence upon vision is ascribed also to constricting articles of neckwear, owing to the impeding of the return column of the blood. Touching the question of type-writing, Dr. Fox advises that, if there is any visual defect, after the prescription of the proper glasses, if round finger-tips or keys are used, a change should be made to a machine having rectangular keys, which, in his experience, are the least hurtful to the eyes. Among the concluding hygienic hints, the following are given: Up to forty years of age the eyes should be bathed daily with cold water. After the fortieth year, the eyes should be douched morning and evening with water as hot as can be borne, followed with cold water.

